# CRISPR Disruption of *BmOvo* Resulted in the Failure of Emergence and Affected the Wing and Gonad Development in the Silkworm *Bombyx mori*

**DOI:** 10.3390/insects10080254

**Published:** 2019-08-19

**Authors:** Honglun Bi, Xia Xu, Xiaowei Li, Yong Zhang, Yongping Huang, Kai Li, Jun Xu

**Affiliations:** 1 College of Life Science, East China Normal University, Shanghai 200062, China; 2 Key Laboratory of Insect Developmental and Evolutionary Biology, Institute of Plant Physiology and Ecology, Shanghai Institutes for Biological Sciences, Chinese Academy of Sciences, Shanghai 200032, China; 3 Department of Biology, University of Nevada, Reno, NV 89557, USA

**Keywords:** *Bombyx mori*, CRISPR/Cas9, *BmOvo*, wing development, gonad development

## Abstract

The domesticated silkworm is an economically important insect that is widely used as a lepidopteran insect model. Although somatic sex determination in the silkworm is well characterized, germline sex determination is not. Here, we used the transgenic-based CRISPR/Cas9 genome editing system to study the function of the *Ovo* gene in *Bombyx mori*. *BmOvo* is the homolog of a factor important in germline sex determination in *Drosophila melanogaster*. *BmOvo* mutants had abnormally shaped eggs that were disordered in the ovarioles, and gonad development was abnormal. Interestingly, wing discs and wings did not develop properly, and most of the mutants failed to eclose. Gene expression analyses by qRT-PCR showed that *BmOvo* gene was highly expressed in the wing disc and epidermis. Genes involved in the WNT signaling pathway and wing development genes *BmWCP10* and *BmE74* were downregulated in the *BmOvo* mutants when compared with wild-type animals. These results demonstrate that the *BmOvo* gene product plays an important role in wing metamorphosis. Thus, this study provides new insights into the multiple functions of *BmOvo* beyond germline sex determination.

## 1. Introduction

The insect OVO protein belongs to the zinc finger protein family, and it regulates many biological processes such as neural tube formation [1], eye maintenance [2], epidermal differentiation [3,4,5], and development of germ cells [3,6,7]. In *Drosophila melanogaster*, the *Ovo* gene encodes two functionally antagonistic isoforms, OVO-A, a transcriptional repressor, and OVO-B, which is a transcriptional activator [8]. The expression of *Ovo* is regulated by the X:A ratio and is required in XX germ cells [9,10]. The genes *Ovo*, *Otu*, and *Sxl* are necessary for female germ cell development in *Drosophila* [11,12], and the *Ovo* gene has been shown to be important for female germline survival and oogenesis [13]. During oogenesis, OVO-B is necessary and sufficient for female germline development [13]. Thus, *Ovo* is responsive to germ cell-autonomous cues in *Drosophila* [14,15,16]. 

The silkworm, *Bombyx mori*, as the foundation of sericulture, is an economically important insect; it is also an important model of lepidopteran species. In recent years, genetic manipulation technologies have been implemented in the silkworm; these include germline transformation methods [17,18] and genome-editing techniques such as transcription activator-like effector nuclease (TALEN) and clustered, regularly interspaced, short palindromic repeat (CRISPR)/Cas9 endonuclease-mediated systems [19,20,21,22,23]. The germline transformation using the *piggyBac* transposon element has also been widely used as a genetic manipulation technology to study gene function in the silkworm [24,25,26]. We combined transgenic methods and the genome-editing CRISPR/Cas9 system to explore the gene function in the silkworm [27,28,29,30].

Four transcript isoforms are expressed from the *Ovo* gene in the silkworm. A previous report showed that *BmOvo-1* is most highly expressed [31]. *BmOvo-1* is highly homologous with *DmOVO-B*. *BmOvo-1* reportedly regulates ovary size, protein synthesis, nutrition transportation, and oviposition number through an RNA interference mechanism [31]. In the present study, we further analyzed the function of *Ovo* in *B. mori*. We found that *BmOvo-1* is highly expressed in the wing disc and epidermis. When we knocked out all of four isoforms of the *BmOvo* gene using the CRISPR/Cas9 system, we found that gonad and wing development were defective, and the mutants did not emerge from the pupae. These data indicate that the *BmOvo* gene is not only crucial for germ cell sex development, but is also important for normal wing development.

## 2. Materials and Methods

### 2.1. Silkworm Strain

A multivoltine, nondiapausing silkworm strain, Nistari, was used for germline transformation and subsequent experiments. Larvae were reared on fresh mulberry leaves under standard conditions [28].

### 2.2. Quantitative Real-Time PCR (qRT-PCR)

For qRT-PCR analyses, total RNA was extracted from the silkworm larvae using Trizol reagent (Invitrogen, Carlsbad, CA, USA) and treated with RNase-free DNAse I (Ambion, Austin, TX, USA), according to the manufacturer’s protocol. cDNAs were synthesized using the Omniscript Reverse Transcriptase kit (Qiagen, Hilden, Germany) in a 20-μL reaction mixture containing 1 μg total RNA. RT-PCR reactions were carried out using gene-specific primers (forward, 5′-GCCCCTTACCGCTCCTTTCG-3′, reverse, 5′-ATCGCCTCCAAGAATCGATG-3′) to amplify a 137-bp fragment of the *BmOvo-1* gene. Another primer pair set (forward, 5′-TCAATCGGATCGCTATGACA-3′, reverse, 5′-ATGACGGGTCTTCTTGTTGG-3′) was used to amplify a 136-bp fragment from the *B. mori* ribosomal protein 49 (*Bmrp49*) as an internal control.

### 2.3. Plasmid Construction

Two small guide RNAs (sgRNAs) targeting sites on the first and third exons of *BmOvo* (GenBank accession number 477588.1) were designed according to the GN_19_NGG rule [32]. Selected sgRNA sequences were examined for potential off-target binding to silkworm genomic sequences using CRISPRdirect (http://crispr.dbcls.jp/) [33]. Moreover, sequences were subjected to BLAST analysis against the silkworm genome to avoid sequences with high homology to non-targeted genes. The binary transgenic system was established by expressing *Cas9* and two sgRNAs in separate plasmids. The plasmid *pBac[IE1-EGFP-SV40-nos-Cas9 -SV40]*, which constitutively expresses *Cas9* under the control of the *nos* gene promoter, has been described by our lab [34]. To obtain a transgenic plasmid constitutively expressing two *BmOvo* sgRNAs, two cassettes of U6:sgRNA scaffold:polIII terminator were constructed using the silkworm U6 promoter [35]. Two U6 promoter sequences were amplified by PCR using primers complementary to the endonuclease restriction enzyme sites NheI and SalI using the silkworm genomic DNA as the template, and sub-cloned into the initial plasmid to generate *pBac[IE1-DsRed-U6-NheI-U6-SalI]*. The two sgRNAs were amplified using the primers Ovo-sgRNA1-F/sgRNA-R and Ovo-sgRNA2-F/sgRNA-R and inserted into the NheI and SalI restriction enzyme sites, respectively, to generate the final plasmid U6-Ovo sgRNA×2. The plasmids were extracted with a Plasmid Midi Kit (Qiagen) according to the manufacturer’s instructions and purified by phenol-chloroform extraction [28]. All primers sequences are listed in Table 1.

### 2.4. Germline Transformation

For silkworm germline transformation, preblastoderm Nistari embryos were microinjected with a mixture of transgenic plasmid and helper plasmids and subsequently incubated at 25 °C in a humidified chamber for 10–12 days until larval hatching. Putative transgenic generation 0 (G0) moths were sib-mated or mated to wild-type (WT) moths and G1 progeny were scored for the presence of the fluorescent marker using fluorescence microscopy (Nikon AZ100, Minato, Tokyo, Japan).

### 2.5. Mutagenesis Analysis

Genomic PCR, followed by sequencing, was carried out to identify *BmOvo* mutant alleles induced by CRISPR/Cas9. The genomic DNA was extracted from larvae at the fifth instar with a DNA extraction buffer, incubated with proteinase K, and purified via phenol:chloroform extraction and isopropanol precipitation, followed by an RNaseA treatment. The PCR conditions were as follows: 98 °C for 2 min, followed by 35 cycles of 94 °C for 10 s, 55 °C for 30 s, and 72 °C for 1 min, followed by a final extension period of 72 °C for 10 min. The PCR products were cloned into the pJET1.2-T vectors (Fermentas, Waltham, MA, USA) and sequenced directly as previous reported [36]. The primers F1 and R1 (Table 1) were designed to detect mutations in the targeted sites. These mutants were photographed to observe the abnormal phenotypes and compare them with the wild type with a digital stereoscope (Nikon AZ100).

### 2.6. Statistical Analysis

Data were analyzed with SPSS 2.0 using the two-tailed *t*-test. *t*-test: *, *p* < 0.05, **, *p* < 0.01, ***, *p* < 0.001. At least three independent replicates were used for each treatment, and the error bars show means ± S.E.M.

## 3. Results

### 3.1. BmOvo-1 Is Highly Expressed in Wing Disc and Epidermis

A previous study evaluated the expression patterns of *BmOvo-1* in a few tissues [31]. To investigate the spatial expression pattern of *BmOvo-1* in detail, qRT-PCR was used to analyze cDNA prepared from the head, fat body, wing disc, epidermis, middle silk gland, posterior silk gland, midgut, testis, and ovary. Tissues were collected from the final instar larvae (the third day of the fifth larval instar, L5D3). qPCR analysis showed that *BmOvo-1* transcripts were detected in all these tissues. This transcript was most abundant in the wing disc and epidermis (Figure S1). This suggested that *BmOvo* might play a role in the development of the wing or epidermis.

### 3.2. CRISPR/Cas9-Mediated Mutagenesis of BmOvo Gene

**Figure 1:**
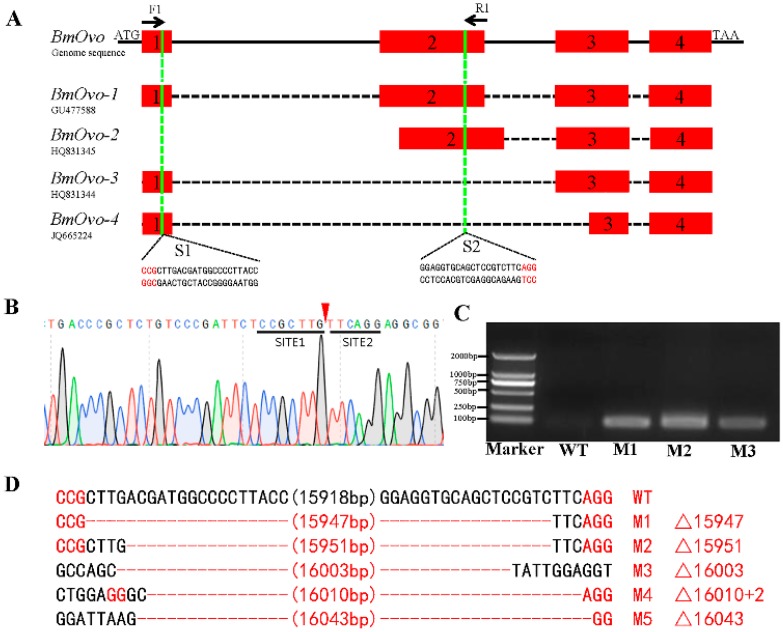
CRISPR/Cas9-induced mutagenesis results in knockout of *BmOvo*. (**A**) Schematic of the targeted region of *BmOvo*. The boxes indicate exons, and the black lines represent introns. The proto spacer adjacent motif (PAM) sequence is shown in red. The sgRNA target sites, S1 and S2, are located on the sense strand within exon 1 and exon 3, respectively. Binding sites for primers F1 and R1 are indicated. The sgRNA target sequence is shown. (**B**) Representative sequencing chromatogram of PCR products from the genome sequencing of *BmOvo* mutants. SITE1 and SITE2 indicate sequences of a portion of each target site sequence. In wild-type insects, the two target sites are separated by about 16 kb, but the target sites are adjacent in mutants where *BmOvo* has been knocked out. (**C**) Genomic PCR detected *BmOvo* deletion mutants. WT, wild type. M1, M2, and M3 are the mutants. (**D**) Sequences of *BmOvo* deletion mutants. The numbers in parentheses indicate the number of base pairs deleted between the two targeted regions relative to the wild-type sequence. The sequence in red indicates the PAM sequence.

To explore the function of *BmOvo* gene *in vivo*, we constructed a binary CRISPR/Cas9 system to knock out the *BmOvo* gene essentially, as previously described [28,34,37]. This system consisted of two transgenic lines, *nos*-Cas9, expressing the Cas9 protein driven by the *nanos* promoter, and U6-sgRNA, expressing sgRNAs driven by U6 promoter (Figure S2A). According to previous reports [31], there are four different transcript isoforms of *BmOvo* gene. These four isoforms all include exon 1 and three include exon 2. sgRNAs were designed to target sites in these exons (Figure 1A). By selecting the red and green fluorescence protein markers, we obtained binary lines that expressed Cas9 and sgRNA, respectively (Figure S2B).

The binary lines *nos*-Cas9 and U6-sgRNA were crossed and double fluorescence G1 individuals were obtained. These animals were subjected to somatic mutagenesis analysis. None of the *BmOvo* transcripts were detected in these lines (Figure 1A). Genome sequencing demonstrated successful deletion of the sequence between the two targeted sites in the *BmOvo* gene (Figure 1B). We used the primers F1 and R1 to detect the wild-type and mutant sequence. No PCR product was obtained from extracts of wild-type insects because the primer binding sites were about 16 kb apart, but PCR products were detected in mutants (Figure 1C,D).

### 3.3. Loss of BmOvo Results in Abnormal Development of Wings and Some Other Organs

**Figure 2:**
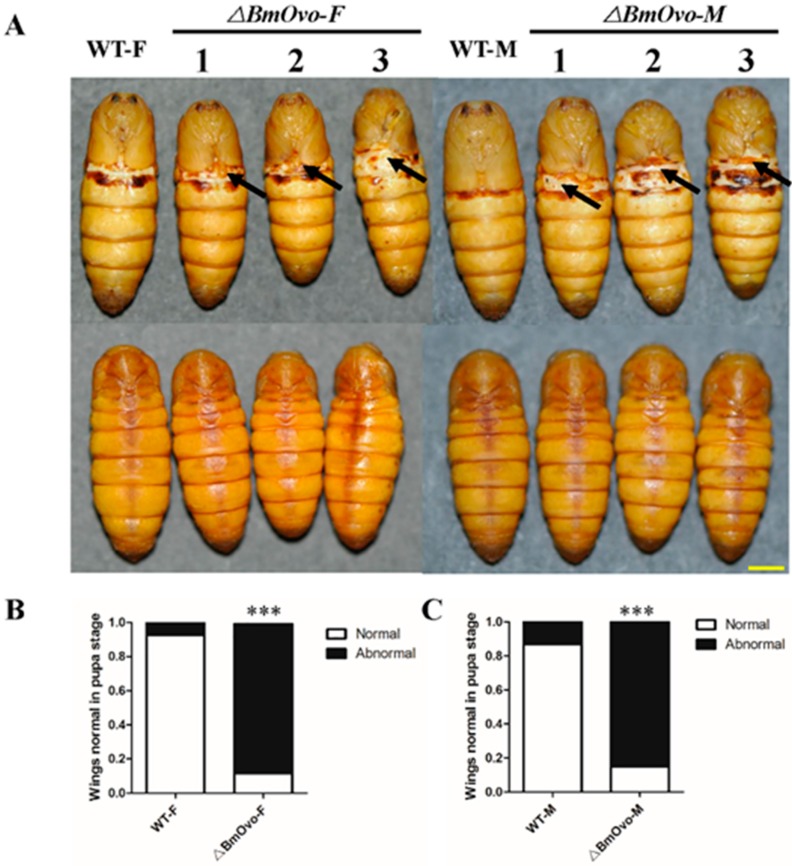
*BmOvo* mutants have abnormal wings in the pupal stage. (**A**) Photographs of wild-type and *BmOvo* mutant female (**left**) and male (**right**) silkworms in the pupal stage. Arrows indicate wing abnormalities. The back sides of the mutants are normal. Scale bar, 2 mm. (**B**) Fraction of female wild-type (n = 100) and *BmOvo* mutants (n = 78) with abnormal wings. Fraction of male wild-type (n = 100) and *BmOvo* mutants (n = 89) with abnormal wings. The asterisks indicate significant differences (** *p* < 0.01 and *** *p* < 0.001) compared with the wild type in the pupae stage determined using a two-tailed *t*-test.

**Figure 3:**
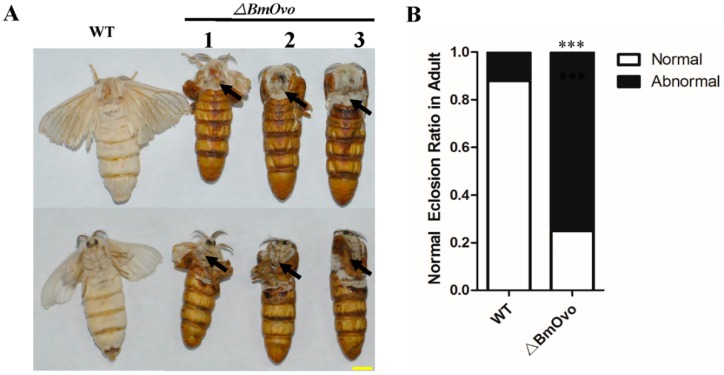
The *BmOvo* mutants do not undergo metamorphosis from the pupal to adult stage. (**A**) Images of wild-type and *BmOvo* mutant animals after the pupal to adult transition. Scale bar, 2 mm. (**B**) The fraction of wild-type (n = 200) and *BmOvo* mutant animals (n = 167) that eclosed normally. The asterisks indicate significant differences (** *p* < 0.01 and *** *p* < 0.001) compared with the wild type in the pupae stage determined using a two-tailed *t*-test.

Morphologically, there were no obvious differences in metamorphosis when *BmOvo* mutants were compared to WT animals in the larval stage. However, we found that pupal and adult wings were twisted and short and that female mutants were shorter than the wild type at the pupal stage (Figure 2A). The wing defect had no sexual bias; 85.9% and 84.2% of the female and male pupae were abnormal, respectively (Figure 2B,C). About 8% and 13% of wild-type female and male pupae, respectively, had a wing defect. Through the dissection, we also detected some abnormalities in the internal organs. The wing disc was atrophic and malformed when compared with the wild type (Figure S3a,a′), and the testes and ovaries of the mutants were smaller than those of the wild type in L5D3 larvae (Figure S3b–c′). For the female moth mutants, we found that the arrangement of eggs in the ovarioles was disordered when compared with the wild type (Figure S3d,d′). As a result of the defective wings, only 25% of mutants eclosed normally, whereas 88% of wild-type animals did (Figure 3). The failed or abnormal metamorphosis of pupa to adult appeared to be due to failure of wing extension from the puparium. These results demonstrate that *BmOvo* plays important roles in reproduction and wing development in *B. mori*.

### 3.4. BmOvo Influences Expression of Genes Involved in Wing Development and Metamorphosis

**Figure 4:**
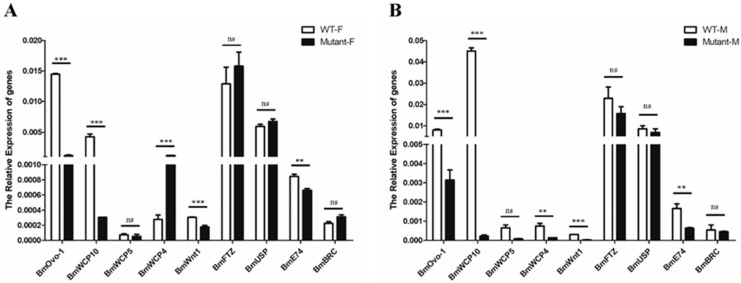
Genes involved in wing development are downregulated in the *BmOvo* mutants. (**A**) Levels of indicated mRNAs in female adult *BmOvo* mutants relative to wild-type levels. (**B**) Levels of indicated mRNAs in male adult *BmOvo* mutants relative to wild-type levels. Three individual biological replicates of real-time PCR were performed. The asterisks indicate significant differences (** *p* < 0.01 and *** *p* < 0.001) compared with the wild type in the adult stage determined using a two-tailed *t*-test.

In order to investigate the mechanism that underlies *BmOvo* phenotypes, we used qRT-PCR to analyze the genes involved in wing development and pupal metamorphosis. Compared with the wild type, the expression of *BmOvo-1* was significantly downregulated in female and male mutants (Figure 4). These results showed the *BmOvo* genes were disrupted successfully. The levels of wing development-related transcripts including wing cuticle protein genes *BmWCP10*, *BmWCP5*, *BmWCP4*, and *BmWnt1* and metamorphosis-related genes *BmFTZ-F1*, *BmUSP*, *BmE74*, and *BmBRC* were quantified. *BmWCP10*, *BmWnt1*, and *BmE74* were downregulated significantly in *BmOvo* mutants when compared to wild-type silkworms, and *BmWCP4* was significantly upregulated in both female and male mutants (Figure 4). There were no significant differences in the expression of metamorphosis-related genes (Figure 4). These results suggest that *BmOvo* affects wing development by influencing the expression of wing development-related genes.

## 4. Discussion

In the present study, we analyzed the function of the *BmOvo-1* gene in vivo by using the CRISPR/Cas9 genome editing system. Due to abnormal wing development, the *BmOvo* deletion mutants failed to eclose from the pupal stage to the adult stage. The wing discs, testes, and ovaries of the *BmOvo* mutants were atrophic and small when compared with the wild type at the L5D3 stage. In mutant female moths, the arrangement of eggs in the ovarioles was disordered when compared with wild-type females. These results demonstrate that the *BmOvo* gene plays an important role in wing development as well as reproduction in *B. mori.* In a previous study, Xue et al. used the RNAi method to inhibit the expression of *BmOvo-1* and *BmOvo-2* and demonstrated that both are involved in reproduction in the silkworm [31]. They did not report wing defects. The 20% decrease in mRNA levels might not be enough to cause wing malformation.

Insects are the most diversified and arguably the most successfully animal group, with more than two million species identified. Insects have invaded every conceivable ecological niche and are the dominant class of organisms on land. Acquisition of flight enabled insects to exploit new habitats and escape from unfavorable environments [38,39]. Wings are also important for predation and mating [40,41]. In butterflies, the wing patterns are variable and are involved in warning, coloration, mimicry, thermoregulation, and mate selection [42,43,44]. 

How insect wings evolved is not well understood, but the study of wings may shed light on insect evolution and development of morphological diversification and speciation [45,46]. Certain pathways and genes are known to influence wing development. Insect metamorphosis is orchestrated by the pathways regulated by the hormone JH and by the ecdysone pathway [47,48], and these pathways also affect wing development [49]. The wing disc forms during the larval stage [50]. In *D. melanogaster*, the wing disc consists of undifferentiated and proliferating cells [51]. In *B. mori*, during the larval stage, the wing discs undergo dramatic morphological changes and evaginate from the body to form the pupal wings [52]. These changes and processes are regulated by insect hormones and hundreds of genes [53]. Wing disc cuticle proteins (WCPs) play crucial roles in the stability of the cuticle layer [54,55]. At least 53 WCPs expressed by *B. mori* are downregulated by JH and upregulated by ecdysone [56,57,58,59].

In *Drosophila*, *Svb* and *Ovo* gene are located in a gene cluster. *Ovo* is required for female germline development and survival [6]. Most of the *Svb* coding sequence is shared with that of the *Ovo* gene. *Svb* encodes a zinc finger transcription factor homologous with *BmOvo* gene products [60]. In *Drosophila*, the *Svb* gene integrates opposing signals emanating from Wg and the EGF-receptor DER, which repress and activate *Svb* transcription, respectively [4]. The transcription factor encoded by *Svb* governs changes in epidermal cell shape and is a major regulator of epidermal development and differentiation [4].

In a previous report, *BmOvo* was implicated in segmentation [61]. When the expression of *BmWnt1* was inhibited in the embryo stage, the expression pattern of *BmOvo* was altered, suggesting that *BmOvo* is regulated by *BmWnt1* [61]. The *Wnt* pathway is involved in embryo development and wing disc formation [25,62]. When we analyzed the expression of the wing disc development-related genes *BmWCP10*, *BmWCP5*, *BmWCP4*, and *BmWnt1* in the *BmOvo* mutants, we found that *BmWCP10*, *BmWCP5*, and *BmWnt1* mRNAs were downregulated compared to levels in wild-type insects. Taken together, our results suggest that the transcription factor encoded by *Ovo* similarly contributes to the wing development in the silkworm.

## 5. Conclusions

In female *BmOvo* created using CRISPR/Cas9, eggs were abnormally shaped and disordered in the ovarioles. In male mutants, gonad development was abnormal. Moreover, knockout of *BmOvo* impaired wing development, and mutant silkworms failed to molt from the pupal stage. Genes involved in the WNT signaling pathway and wing development genes, BmWCP10 and BmE74, were downregulated in the *BmOvo* mutants when compared with the wild-type animals, suggesting that the transcription factor encoded by *BmOvo* is important for wing metamorphosis. The analyses described here demonstrate that BmOvo has functions in addition to those important for germline sex determination.

## Figures and Tables

**Table 1:** Primers used in this work.

Primer Name	Primer Sequence (5′ to 3′)	Primer Purpose
Ovo-sgRNA1-F	TATCGTGCTCTACAAGTGGTAAGGGGCCATCGTCAAG GTTTTAGAGCTAGAAATAG	Preparation of sgRNA template
Ovo-sgRNA2-F	TATCGTGCTCTACAAGTGGAGGTGCAGCTCCGTCTTC GTTTTAGAGCTAGAAATAG	Preparation of sgRNA template
sgRNA-R	TAGATATCAAGCTGCTAGAAAAAAAAGCACCGACTC GGTGCC	Preparation of sgRNA template
F1	ATGCCGAAAATCTTCTGGATTAAG	Detection of mutations
R1	GTTTTTGGTTGATGGACCGAGTGT	Detection of mutations
Ovo-1-qF	GCCCCTTACCGCTCCTTTCG	qRT-PCR
Ovo-1-qR	ATCGCCTCCAAGAATCGATG	qRT-PCR
RP49-qF	TCAATCGGATCGCTATGACA	qRT-PCR
RP49-qR	ATGACGGGTCTTCTTGTTGG	qRT-PCR
WCP10-qF	TGGAGCACGCCTTCATATCA	qRT-PCR
WCP10-qR	GGACGGTGTAAACTTTGCCA	qRT-PCR
WCP5-qF	GCAGCCCCTTTGATTCAACA	qRT-PCR
WCP5-qR	CGTGTTGGGACTTGTGATCG	qRT-PCR
WCP4-qF	AGTCCACGAGGCTTCTTC	qRT-PCR
WCP4-qR	CCTTGCGGAATGAACCA	qRT-PCR
Wnt1-qF	CAGGGAATTCGTTGATACCG	qRT-PCR
Wnt1-qR	TCATCCAGCAAGTCTTCACG	qRT-PCR
FTZ-F1-qF	ATGCGTCGCCGAAAGAGCCT	qRT-PCR
FTZ-F1-qR	ATTGCGACCACCGCGCATAC	qRT-PCR
USP-qF	ACACTTCGGGCAGCTAGAA	qRT-PCR
USP-qR	TCCGCGAGTCTACGTTCTCT	qRT-PCR
E74-qF	GCACAAGAACAAGCCAGACA	qRT-PCR
E74-qR	GTCGATCTCGACGATGTCCT	qRT-PCR
BRC-qF	AAAGGCCTCCCTGAAGAGAC	qRT-PCR
BRC-qR	CGCGACTTGTGGTAGGTGTA	qRT-PCR

## Supplementary Materials

The following are available online at https://www.mdpi.com/2075-4450/10/8/254/s1, Figure S1: The expression of BmOvo-1 in head, fat body (FB), wing disc (WD), epidermis (Epi), middle silk gland (MSG), posterior silk gland (PSG), midgut (MG), and gonad at L5D3. RNA was extracted and reverse transcribed to cDNA and analyzed by real-time PCR. Three individual biological replicates of real-time PCR were performed., Figure S2: Transgenic plasmid construction and identification of BmOvo knock-out lines. (A) Schematic of the binary transgenic CRISPR/Cas9 system, which involves one line for expression of the full-length Cas9 ORF driven by the nos promoter and another line for U6 promoter-driven expression of two sgRNAs. These two lines also contain the reporter genes EGFP and DsRed, respectively, under the control of the IE1 promoter. (B) Parental transgenic strains expressing Cas9 or sgRNA were established. Somatic mutations were induced in the F1 founder animals following crosses of these two strains. Red and green fluorescence in the entire body confirmed the presence of the appropriate transgene constructs. Scale bars, 1 mm., Figure S3: The BmOvo mutants showed abnormal wing discs, disorder eggs in the ovarioles, small testes and ovaries. (a) Wild-type and (a′) mutant wing disc at L5D3-stage. The black arrows indicate the atrophic and malformed region of wing disc in BmOvo mutant. (b) Wild-type and (b′) mutant testes at L5D3 stage. (c) Wild-type and (c′) mutant ovaries at L5D3 stage. (d) Wild-type and (d′) mutant ovarioles in adults. Scale bars, 0.5 mm.
