# Supplementary figures and images for: CRISPR Disruption of BmOvo Resulted in the Failure of Emergence and Affected the Wing and Gonad Development in the Silkworm Bombyx mori
Source: Insects. 2019 Aug 19;10(8):254. doi: 10.3390/insects10080254 (PMC6723145; doi:10.3390/insects10080254)

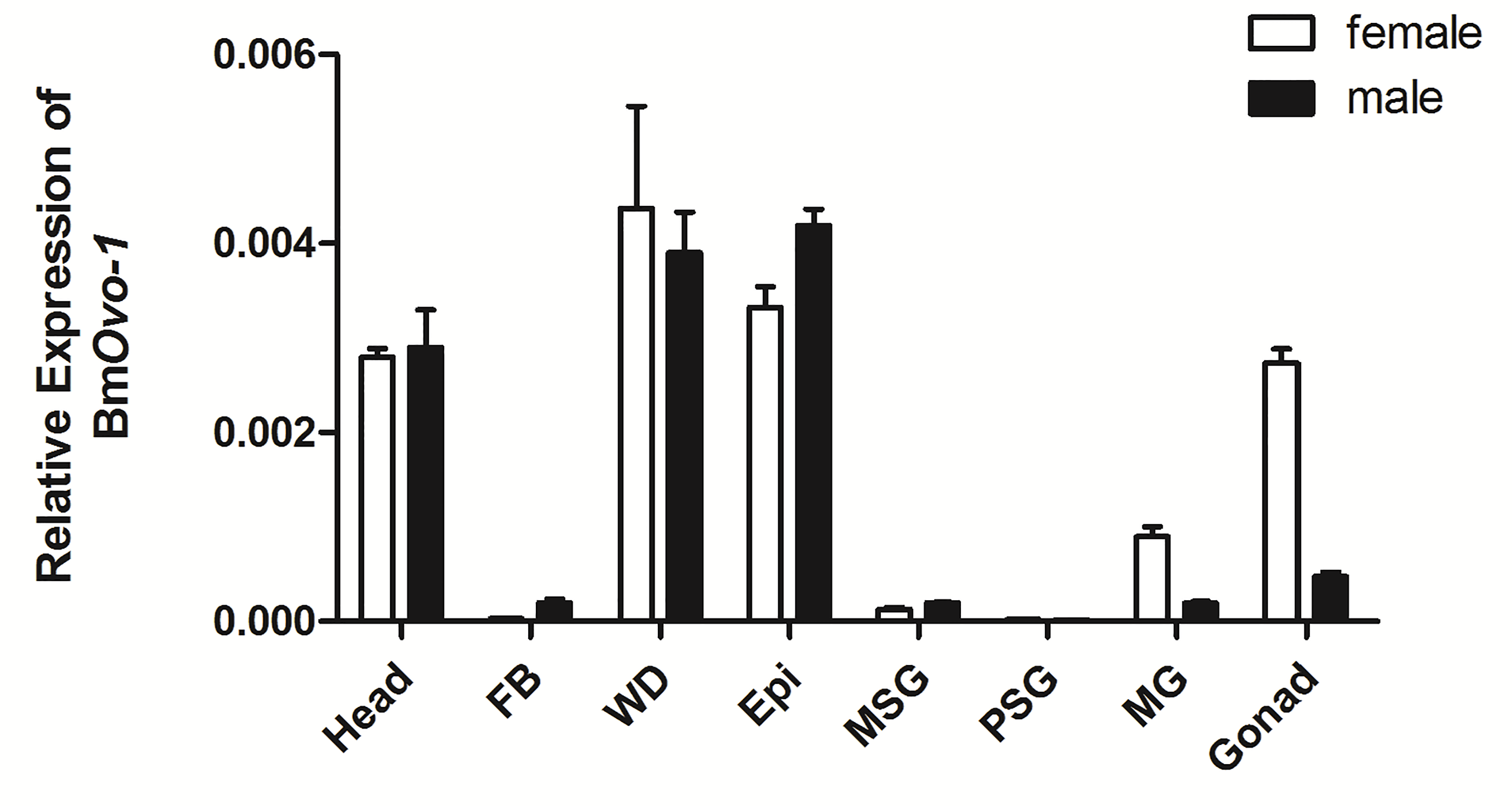

Supplement: Supplementary file 1 [file insects-10-00254-s001.zip › Supplementary Files/fig s1.tif]

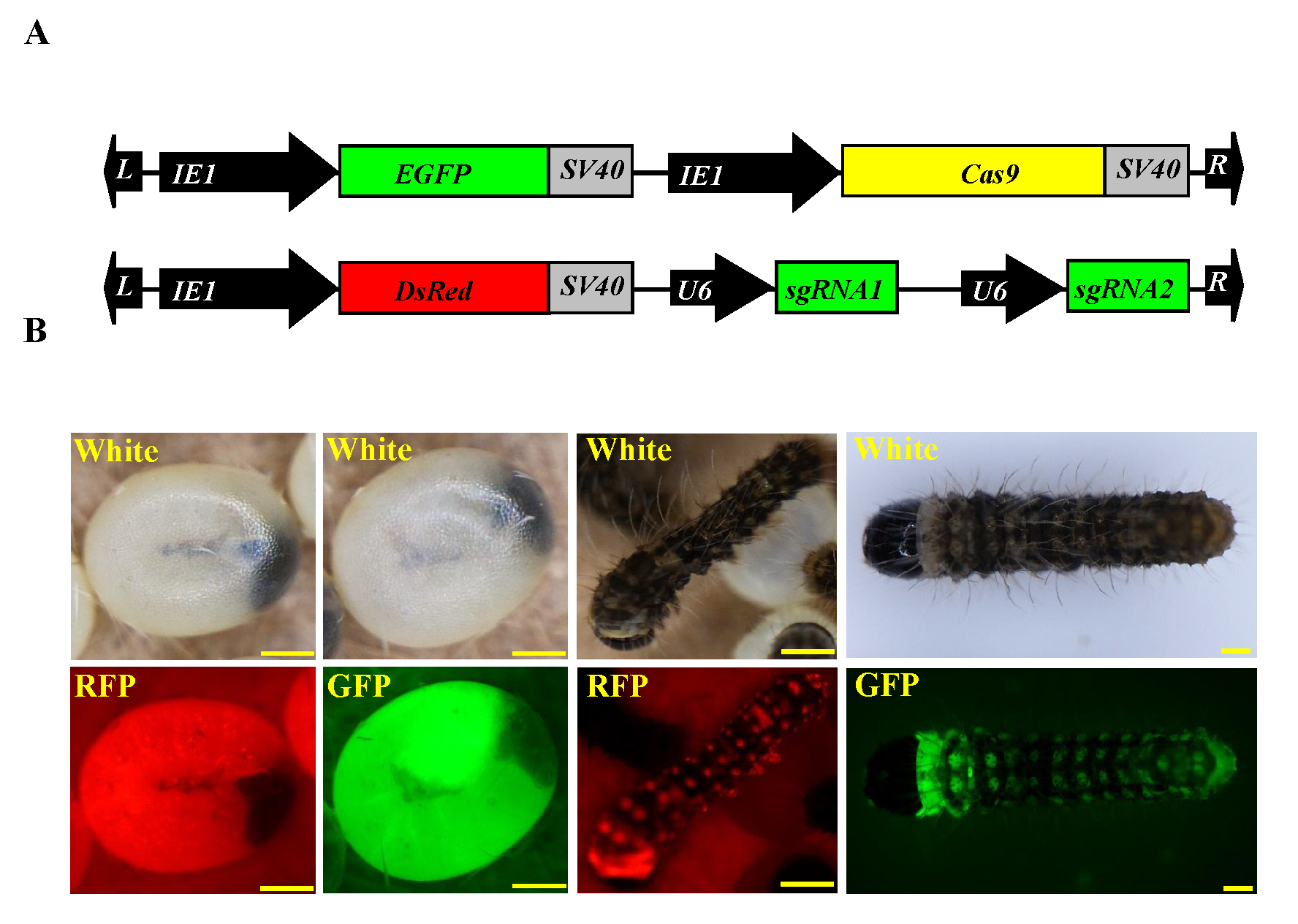

Supplement: Supplementary file 1 [file insects-10-00254-s001.zip › Supplementary Files/fig s2.tif]

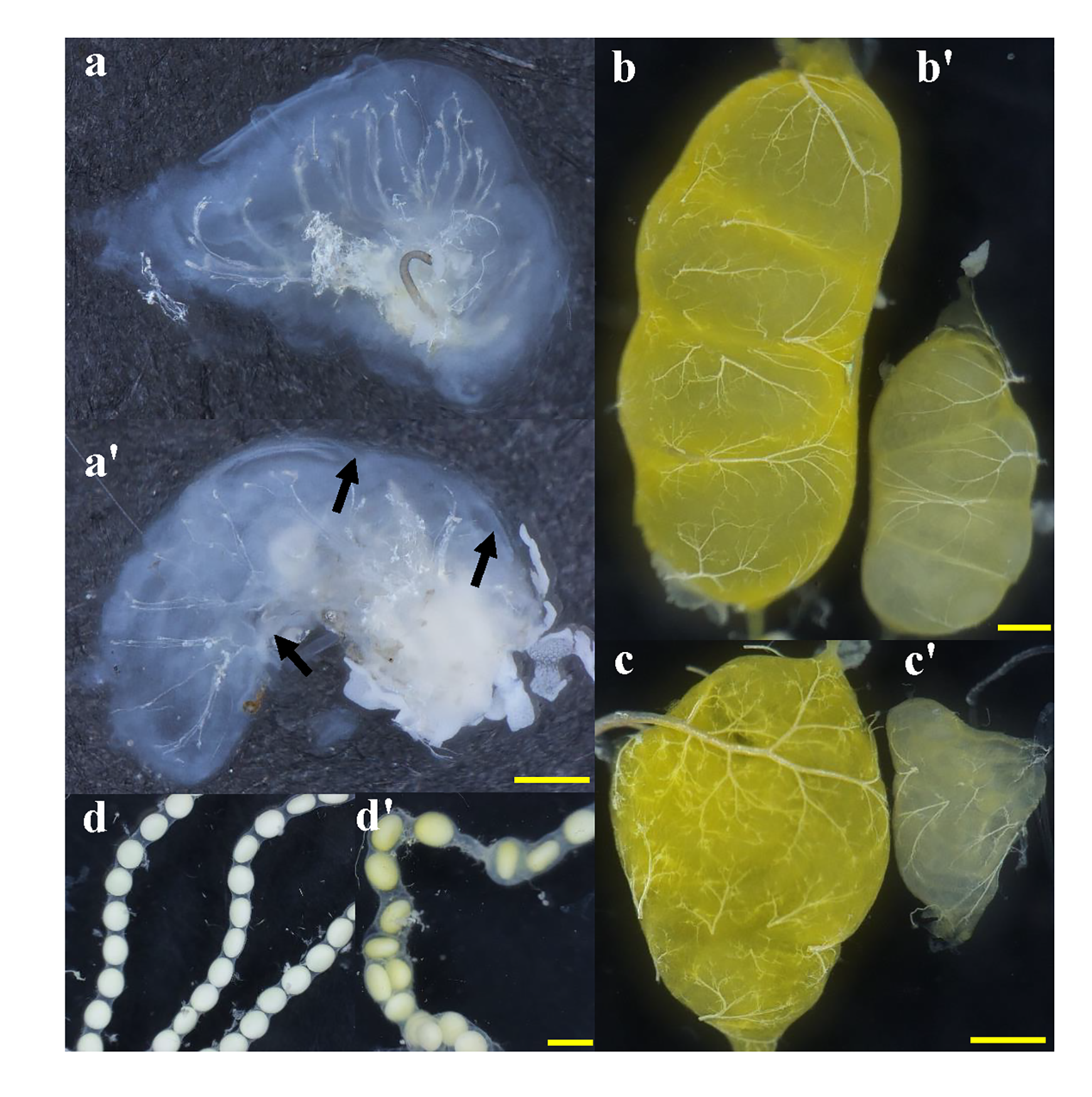

Supplement: Supplementary file 1 [file insects-10-00254-s001.zip › Supplementary Files/fig s3.tif]
